# Fatal Suicidal Intoxication with Pentoxifylline Complicated by Cardiovascular Disorders

**DOI:** 10.3390/toxics10080447

**Published:** 2022-08-03

**Authors:** Jacek Sein Anand, Marek Wiergowski, Marek Roman Wiśniewski, Monika Kosmowska, Marzena Kata, Mateusz Kacper Woźniak

**Affiliations:** 1 Department of Clinical Toxicology, Faculty of Health Sciences with the Institute of Maritime and Tropical Medicine, Medical University of Gdansk, M. Sklodowskiej-Curie 3a Str., 80-210 Gdansk, Poland; jacek.anand@gumed.edu.pl (J.S.A.); marek.wisniewski@gumed.edu.pl (M.R.W.); 2 Pomeranian Centre of Toxicology, Kartuska 4/6 Str., 80-104 Gdansk, Poland; 3 Department of Forensic Medicine, Faculty of Medicine, Medical University of Gdansk, M. Sklodowskiej-Curie 3a Str., 80-210 Gdansk, Poland; monika.kosmowska@gumed.edu.pl (M.K.); marzena.kata@gumed.edu.pl (M.K.); mateusz.wozniak@gumed.edu.pl (M.K.W.)

**Keywords:** pentoxifylline poisoning, overdose, suicide, bioanalysis, liquid chromatography coupled with tandem mass spectrometry

## Abstract

Pentoxifylline is a xanthine derivative used in vascular disorders that is recognized as a safe drug for patients. The paper describes a rare case of fatal and suicidal pentoxifylline poisoning in an 82-year-old man with multiple preexisting diseases (arterial hypertension, coronary artery disease, type 2 diabetes, and intermittent claudication). The patient was admitted to the clinical toxicology unit approximately 2 h after the overdose and died 36 h after the admission despite intensive care. Multiple arterial blood gas analyses and other laboratory tests were performed during the hospitalization and are reported in the paper. Postmortem examination of the biological material was carried out with the use of histopathological techniques. The toxicological studies using chromatographic techniques coupled with mass spectrometry showed that postmortem blood levels of pentoxifylline have been found in the range which is described in the available literature to be toxic and lethal. The analysis of test results and clinical data showed that the patient died as a result of increasing circulatory and respiratory failure, complicated by disorders of the acid-base and electrolyte balance (respiratory alkalosis, concomitant lactic acidosis, and hypokalemia), hyperglycemia, and coagulation disorders.

## 1. Introduction

Pentoxifylline is a synthetic xanthine derivative used in the treatment of peripheral blood supply disorders of lower extremities, and in the thrombotic and occlusive blood vessel diseases, as well as in the diabetic neuropathy and retinopathy. It is also used in the hearing and balance disorders caused by ischemia and in circulatory disorders within the eyeball. Pentoxifylline improves the rheological properties of blood by increasing the concentration of prostaglandin I2 (PGI2, prostacyclin), inhibiting phosphodiesterase, and increasing the concentrations of adenosine triphosphate (ATP), cyclic adenosine monophosphate (cAMP), and cyclic guanosine monophosphate (cGMP) in erythrocytes. The reduction in blood viscosity is due to increased elasticity of erythrocytes.

The biological half-life of pentoxifylline is approximately 0.4–1.0 h and the volume of distribution is 3.3–5.0 L/kg [1]. The drug is administered to adults at a dose of approximately 800–1200 mg/day for around 8 weeks. Orally administered pentoxifylline is absorbed completely, reaching the peak serum concentration after about 2–3 h.

Most of pentoxifylline is eliminated with urine as metabolites, a few of which are active and only about 4% is excreted with the feces. Pentoxifylline does not bind to blood proteins. In people with renal insufficiency, or the elderly, there is a possibility of accumulation of pentoxifylline metabolites in the body.

Pentoxifylline increases the effect of drugs that lower blood pressure and dilate blood vessels. In adults, an overdose of the drug may cause flushing, low blood pressure, convulsions, sleepiness, loss of consciousness, hyperthermia, fever, and agitation. Treatment of pentoxifylline poisonings is mostly symptomatic.

## 2. Case Report

In July 2021, an 82-year-old patient (67 kg body weight) was admitted to the Pomeranian Centre of Toxicology in Gdansk, due to a suicidal intoxication with about 28 g of pentoxifylline (70 prolonged-release tablets containing 400 mg of pentoxifylline each, dose ingested: 417.9 mg/kg b.w.). According to eyewitnesses, about an hour after overdosing the patient developed profuse vomiting with brown content that looked like “coffee grounds” and quantitative disturbance of consciousness in the form of somnolence. These symptoms were the reason why the patient’s family called for medical assistance. During the transport to the hospital, the patient vomited with similar content several times. The medical history showed that the patient had been treated for depressive disorder. A year before the overdose, he was hospitalized in a psychiatric unit after a suicidal attempt involving self-mutilation. The patient had a medical history of arterial hypertension, stable coronary artery disease, chronic atrial fibrillation, type 2 diabetes, and intermittent claudication.

## 3. Hospitalization

### 3.1. 2 h after Overdose

The patient was admitted to the toxicology unit approximately 2 h after the overdose. On admission, the patient was conscious, with limited verbal-logical interaction, confused, and periodically agitated. In the toxicology unit, the patient vomited three times with brown fluid containing fragments of tablets and food. In the physical examination: blood pressure was 116/45 mm Hg, heart rate was irregular, with ventricular rate of 60–70 bpm. (atrial fibrillation), sat. O_2_ 98%. Physical examination did not reveal any clinically significant abnormalities, except atrial fibrillation. Laboratory tests performed on admission showed: WBC 15.47 G/L, neutrophils 9.35 G/L, lymphocytes 4.84 G/L, monocytes 1.08 G/L, glucose 294 mg/dL, lactate 11.7 mmol/L, K 2.8 mmol/L, BE −4.0 mmol/L; otherwise no clinically significant abnormalities. Arterial blood gases revealed respiratory alkalosis with concomitant metabolic acidosis. The results of subsequent arterial blood gas tests and selected biochemical parameters are presented in Table 1.

The serum ethanol concentration on admission was <0.1 g/L (negative). The electrocardiography (ECG) revealed: atrial fibrillation with ventricular rate of approximately 60–70 bpm., right bundle branch block. The patient was administered intravenous infusion of crystalloids, potassium supplementation and insulin by subcutaneous injections.

### 3.2. 12 h after Overdose

Approximately 12 h after the overdose, the patient was conscious, with limited logical interaction and periodically confused. The arterial blood pressure was 176/89 mmHg, the heart rate was irregular, approximately 80–100 bpm., oxygen saturation (sat. O_2_) 95%. The fluid balance was negative, the patient received 2500 mL of IV crystalloids and spontaneous diuresis was 3700 mL. Diffuse subcutaneous hemorrhages were noticed on the patient’s skin in the abdominal area. Laboratory tests revealed: International Normalized Ratio (INR) 1.41, fibrin degradation product (D-dimer) 4088 ng/mL FEU, white blood cell (WBC) 36.37 G/L, neutrophils 30.76 G/L, lymphocytes 4.84 G/L, monocytes 3.87 G/L; otherwise, no clinically significant abnormalities. Due to the increase of the metabolic acidosis, the patient was given a bolus of 160 mEq of sodium bicarbonate (NaHCO_3_) IV, followed by 20 mEq NaHCO_3_ in every 500 mL of crystalloids. No significant abnormalities were observed in the lung ultrasound examination. The control ECG showed: atrial fibrillation with ventricular rate of approximately 100–120 bpm, right bundle branch block, QTc 469 ms. Due to the elevated blood glucose values (maximum 559 mg/dL), continuous IV insulin infusion was used.

### 3.3. 20 h after Overdose

Due to the decrease in oxygen saturation (sat. O_2_ < 90%), as well as the increase in respiratory effort, the patient was intubated, and mechanical ventilation was applied approximately 20 h after the overdose. Due to the persistent metabolic acidosis, higher than standard minute ventilation was used.

### 3.4. 24 h after Overdose

The patient had a gradual deterioration in cardiovascular function approximately 24 h after the overdose. It was necessary to use a continuous infusion of norepinephrine. Despite this, the patient’s condition continued to deteriorate. Oliguria was noted, followed by anuria.

### 3.5. 36 h after Overdose

The patient experienced a sudden cardiac arrest in the mechanism of asystole 36 h after the pentoxifylline overdose. Cardiopulmonary resuscitation was performed, but was not successful and the patient was pronounced dead after 30 min of resuscitation efforts (on the 27 July 2021).

## 4. Autopsy Results

The autopsy was performed in the Department of Forensic Medicine at the Medical University of Gdansk (on 30 July 2021), during which it was found: postmortem changes (autolysis); a presence of four softening tablets within the duodenum; petechiae under the pulmonary pleura and under the epicardium; chronic lesions: adhesions in the right pleural cavity; cardiac muscle hypertrophy; generalized atherosclerosis, including coronary atherosclerosis, of high intensity; a scar in the muscle of the ventricular septum of the heart and at the apex of the heart; fatty liver; calcification of the spleen capsule; traces of medical intervention, in the form of long-established implantation of stents into the right and left coronary arteries; bilateral, symmetrical fracture of the ribs, without hemorrhages in soft tissues in the vicinity of fractures (state after resuscitation); and traces of numerous punctures in typical places.

The results of histopathological examinations: the brain—with slight hyperemia and in places with a visible ethmoid state; lungs—pulmonary parenchyma with features of significant congestion and extensive fields of edema; and heart—with moderate interstitial and perivascular fibrosis and slight perivascular steatosis. In addition, a visible post-infarction scar and single, small scars separating the muscle fibers (*cicatrix post infarctus et cicatrices parvorum myocardii*), cardiomyocytes with markers of hypertrophy from moderate to significant; liver—with steatosis of approximately 30% of hepatocytes of mixed, mainly large-droplet character, moderate periportal fibrosis, and not very abundant depletion of lymphocytes in the gate spaces, and slight cholestasis also visible; kidneys—apart from moderate hyperemia with severe arteriolosclerosis, pancreas with almost fully autolysed parenchyma, quite abundantly overgrown with adipose tissue; and spleen—with the same proportions between white and red pulp, with clear hyperemia.

## 5. Analysis of Post-Mortem Biological Material

Toxicological analyses of postmortem biological material (blood and urine) were carried out to detect pharmacologically active substances, including medical drugs taken permanently by the patient (according to the information provided by family members): Glucophage (metformin), Gliclade (gliclazide), Trifas COR (torasemide), Prestarium (perindopril), Atorvasterol (atorvastatin), Nebilet (nebivolol), and Polfilin Prolongatum (pentoxifylline).

In addition, the analyses included drugs administered during the hospitalization in the form of: Relanium (diazepam), Metoclopramide (metoclopramide), Actrapid (human insulin), Ondansetron (ondansetron), Augmentin (amoxicillin and clavulanic acid), Levonor (norepinephrine), Fragmin (dalteparin), Midanium (midazolam), Fentanyl (fentanyl), Pancuronium (pancuronium), and Pyralgin (metamizole). All chemicals and standards, as well as analytical equipments, used in the study were described in the Electronic Supplementary Material (ESM).

Initially, for the qualitative analysis purpose, liquid biological postmortem samples of the patient (blood, urine, and vitreous humors) were prepared by liquid–liquid extraction (LLE) both in acidic (pH 3) and base (pH 11) conditions by adding appropriate buffer. The 20 g of tissue samples (kidney, brain, and liver) were extracted using EXtrelut^®^ NT 20 (Merck Millipore, Darmstadt, Germany) by running in-house-developed methods. After the step of extraction, the instrumental methods were applied as follows: high-performance liquid chromatography coupled with ultravio-let-visible and diode array detector (HPLC-UV/VIS-DAD) and gas chromatography coupled with mass spectrometry (GC-MS). Extracts were injected into GC-MS system in SCAN mode. Analyses were performed in conditions as follows: Phenomenex “Zebron ZB-5 MS” capillary column (30 m length, 0.25 mm internal diameter, 0.25 mm film thickness), injector temperature 280 °C, oven temperature programmed from 50 to 285 °C; carrier gas-helium; and ion scan analysis in the m/z 35–380 range. The results were compared with the NIST/EPA/NIH, SWGDRUG and Cayman spectral libraries, which contain most of the pharmacologically active substances. Subsequently, the same extracts were analyzed by the HPLC-UV/VIS-DAD system. The analyses were carried out using a monolithic column filled with octadecyl phase-modified silica gel and a mobile phase, which included an aqueous solution of phosphoric acid (0.01%, *v*/*v*) and acetonitrile; UV detection for wavelengths 220 and 256 nm, DAD detector in the range from 190 nm to 370 nm. The results of the chromatographic analysis were compared with the library of ‘UV Spectra of Toxic Compounds’, which contains 2682 UV spectra of toxic and pharmacologically active substances and their metabolites, developed by Pragst and colleagues. The above-described in-house-developed combined HPLC and GC-based procedure allows for qualitatively analysis most of pharmacologically active substances and their metabolites.

In order to verify the qualitative tests and carry out a quantitative analysis of the detected substances and medical drugs taken permanently by the patient (mentioned above), the research was continued using the liquid chromatography technique coupled with tandem mass spectrometry (LC-MS/MS) working in the positive ionization mode by electrospray (ESI+), the detection was carried out in the mode of monitoring selected fragmentation reactions (MRM), while the GC-MS technique worked in the electron ionization (EI) mode and the detection was carried out in the selected ion monitoring (SIM) mode. As the analyzed compounds belong to different chemical groups (differing in physico-chemical properties), it was necessary to use different extraction methods, i.e., LLE and precipitation of proteins with acetonitrile, under different environmental conditions (pH) using different solvents. The calibration of the analytical equipment was performed using the internal standard method with matrix mapping.

Additional information on chromatographic and extraction conditions as well as validation parameters of methods used for quantification were presented in the electronic supplementary material (see ESM). Only selected data were included in view of fact that presented methods are used in our laboratory for routine toxicological analysis. The applied tests confirmed the patient’s intake of pharmacologically active substances by detecting the parent substances and their metabolites used both before and during hospitalization (results of postmortem tests of blood and urine in Table 2).

To verify distribution of substances which were taken by the patient and those administered during the hospitalization, other biological specimens obtained during autopsy were qualitatively analyzed by above-described methods. Qualitative results in additional biological specimens were as follows: in the gastric contents—pentoxifylline, acetaminophen (paracetamol), 4-MAA (metamizole metabolite), metronidazole, lidocaine; in a tablet taken from the duodenum—pentoxifylline; in the vitreous humour—caffeine, pentoxifylline and metronidazole; in the kidney—the presence of caffeine and pentoxifylline; in the brain—caffeine and pentoxifylline; in the liver—caffeine and pentoxifylline; and in the bile—caffeine and pentoxifylline.

## 6. Discussion

Pentoxifylline overdoses are relatively rare. The review of literature produced only three descriptions of suicidal attempts with this medication. Sznajder et al. reported a case of a 22-year-old woman who ingested approximately 4–6 g of pentoxifylline in a suicidal attempt. The clinical course was complicated by a second-degree atrioventricular block with the ventricular rate of approximately 30 bpm. Bradycardia resolved after administration of 1 mg of atropine IV; however, a first-degree atrioventricular block persisted for another 16 h. Other symptoms were persistent nausea and vomiting, anxiety, and agitation. Hypokalemia (2.7 mmol/L) was observed in the laboratory studies [2].

Suarez-Peñaranda and al. described a case of a 54-year-old man, who ingested approximately 20–24 g of pentoxifylline. The patient was admitted to the hospital approximately 2 h after the overdose and died within 24 h due to a circulatory collapse. Hypokalemia and metabolic acidosis were observed in the laboratory studies. A ventricular tachyarrhythmia with an atrioventricular dissociation was noticed in the ECG. There was also an episode of atrial fibrillation which resolved after the administration of amiodarone. Pentoxifylline concentration in the postmortem blood sample was 32.5 µg/mL [3].

Eden et al. reported a case of a 42-year-old woman (body weight 69 kg), who ingested approximately 20 g of pentoxifylline. The patient was found unconscious after approximately 7 h from the overdose. The patient was intubated on site and mechanical ventilation was applied. Continuous IV infusion of norepinephrine was also started on site due to the low blood pressure values. The arterial blood gases test on admission to the hospital showed a severe metabolic acidosis: pH 6.97, HCO_3_ 9.2 mmol/L, lactate 16 mmol/L, BE −19 mmol/L. The patient was also oliguric; therefore, the decision was made to perform a 4 h intermittent hemodialysis procedure (blood and dialysate flows were both 300 mL/min). During hemodialysis, improvement of arterial blood pressure, return of diuresis and correction of the metabolic acidosis were achieved. The analysis of blood samples obtained before and after the hemodialysis revealed a significant reduction of the blood pentoxifylline concentrations from 23 to 8.7 mg/L. Further reduction of the blood pentoxifylline concentration to unmeasurable values was seen within 25 h from the start of the hemodialysis. The patient was discharged from the intensive care unit after 36 h of treatment [4].

Based on all the reported cases, it seems that the main symptoms of pentoxifylline overdose include: metabolic acidosis, hypokalemia, arrhythmias (both brady- and tachycardia have been reported), cardiovascular collapse, oliguria, and disturbed consciousness (agitation, delirium, and coma).

Treatment is usually symptomatic and includes: fluid therapy, NaHCO_3_ supplementation, and norepinephrine infusion. In the case of massive overdoses, gastric lavage should be considered. Extracorporeal elimination is currently not recommended; however, based on the report by Eden et al., it seems worthy of consideration in patients with a severe metabolic acidosis or kidney dysfunction. In patients with adequate diuresis renal elimination seems to be sufficient.

In the case reported by us, the pH values were within the normal range for most of the clinical course. Initially clinical improvement was achieved with supportive treatment and diuresis was spontaneous and adequate. The fatal result of the overdose was likely largely influenced by the patient’s age and multiple comorbidities.

Severe lactic acidosis is also a feature of “metformin-associated lactic acidosis” (MALA). In the case reported by us, there was no suspicion of an intentional metformin overdose, but before the suicidal overdose with pentoxifylline, the patient was taking 500 mg of metformin twice-daily. The postmortem blood metformin concentration was within the therapeutic range and well below the 9.9 mg/L threshold for MALA reported by Bennis et al. [5]. However, since metformin concentrations were not measured on admission and during treatment, it cannot be excluded that they were significantly higher than postmortem. The patient’s declining renal function makes the assessment of the pharmacokinetics of metformin unfeasible. The lactate levels measured during treatment were lowering toward the normal range, so it is not likely that metformin was a principal factor influencing the fatal outcome of treatment. 

Pentoxifylline concentrations in blood and urine were not measured antemortem due to the limitations of the clinical toxicological laboratory and no samples were preserved for later testing. The clinical significance of blood pentoxifylline concentration is very limited due to the scarcity of data on acute pentoxifylline overdoses in humans. Currently there is no association between blood concentrations of pentoxifylline and treatment of overdose which is symptomatic.

As a result of tests of biological material collected from the patient’s corpse, the presence of 19 pharmacologically active substances and their metabolites was detected, some of these medical drugs were administered during hospitalization. Quantitative analysis showed the presence of pentoxifylline in blood and urine in the range reported in the literature for toxic and lethal concentrations (respectively in Table 3 and Table 4) [1,6,7].

## 7. Conclusions

Based on the clinical data and the postmortem examinations, it can be concluded that the patient died as a consequence of the intentional pentoxifylline overdose, which caused the deteriorations of cardiovascular and respiratory function with concomitant disorders of acid-base and electrolyte imbalance (respiratory alkalosis, lactic acidosis, and hypokalemia), hyperglycemia, and coagulation disorders. Numerous preexisting conditions (arterial hypertension, coronary artery disease, chronic atrial fibrillation, type 2 diabetes, and intermittent claudication) were additional negative factors. The direct mechanism of death was a cardiac arrest due to the conduction disorders in the heart caused by all of the disorders mentioned above. The forensic toxicological studies showed the values of postmortem blood concentrations of pentoxifylline within the range which is considered toxic or lethal.

## Figures and Tables

**Table 1:** Results of arterial blood gas tests and selected biochemical parameters in the course of the pentoxifylline overdose.

Time from Overdose	2 h	12 h	16 h	20 h	24 h	28 h	32 h
pH	7.522	7.35	7.428	7.475	7.387	7.45	7.32
pCO_2_ * (mmHg)	20.1	14.8	22.2	25.6	22.4	21.8	28.1
pO_2_ (mmHg)	110	94.3	65	134	169	187	50.4
HCO_3_^−^ (mmol/L)	16.4	7.9	14.4	18.6	13.1	14.9	14.1
BE (mmol/L)	−4.0	−15.6	−7.8	−4.4	−9.7	−6.5	−10.8
Na^+^ (mmol/L)	137	146	145	154	152	154	138
K^+^ (mmol/L)	2.8	3.7	3.4	3.2	4.3	3.6	6.0
Cl^−^ (mmol/L)	103	121	115	122	125	126	115
Ca^2+^ (mmol/L)	1.09	1.26	1.19	1.16	1.24	1.20	1.15
lactate (mmol/L)	11.7	11.5	10.6	8.2	8.4	9.1	7.7
glucose (mg/dL)	294	424	559	322	317	277	571

* pCO_2_—partial pressure of carbon dioxide, pO_2_—partial pressure of oxygen, HCO_3_^−^—bicarbonate, BE—base excess, Na^+^—sodium cations, K^+^—potassium cations, Cl^−^—chloride anions.

**Table 2:** Results of toxicological analyses of postmortem biological specimens (blood and urine) carried out by the LLE-LC/MS-MS-ESI-MRM method (medical drugs with metabolites administered during hospitalization are marked in italics).

No	Substance	Concentration, µg/mL	
		Blood	Urine
1	Pentoxifylline	10	22
2	Metformin	2.4	106
3	Acetaminophen (paracetamol)	<LOQ *	3.8
4	Metronidazole	<LOQ	0.92
*5*	*4-MAA (metamizole metabolite)*	*18*	*16*
6	Lidocaine	<LOQ	0.31
*7*	*Metoclopramide*	*0.052*	*0.54*
8	Quetiapine	<LOQ	<LOQ
9	Torasemide	<LOQ	Not detected
10	Escitalopram	0.047	0.19
11	Atorvastatin	0.084	Not detected
*12*	*Diazepam*	*0.18*	*0.065*
*13*	*Oxazepam*	*<LOQ*	*0.004*
*14*	*Nordazepam*	*0.028*	*0.016*
*15*	*Temazepam*	*0.006*	*0.010*
*16*	*Midazolam*	*0.006*	*<LOQ*
*17*	*Fentanyl*	*0.003*	*<LOQ*
18	Tramadol	0.048	0.27
19	Zopiclone	0.012	0.069

* <LOQ means below the limit of quantification of the method. LOQ values: 2.5 µg/mL for acetaminophen; 0.5 µg/mL for metronidazole, lidocaine and torasemide; 0.002 µg/mL for quetiapine, fentanyl, midazolam and oxazepam.

**Table 3:** Reference values of pharmacologically active substances in blood compared to concentrations determined in patient’s blood collected postmortem (medical drugs with metabolites administered during hospitalization are marked in italics) [1,6,7].

No	Substance	Concentration in BLOOD, µg/mL			
		Therapeutical (Reference)	Toxic (Reference)	Lethal (Reference)	Patient (after Autopsy)
1	Pentoxifylline	0.06–5.4	8.7–51	6.3–11	10
2	Metformin	1.0–4.0	45–70	75–110	2.4
3	Acetaminophen (paracetamol)	5–26	30–300	160–1280	<2.5
4	Metronidazole	3–20	2	n/a	<0.5
*5*	*4-MAA (metamizole metabolite)*	*4–11*	*20*	669 *	*18*
6	Lidocaine	0.3–5	8–12	12–44	<0.5
*7*	*Metoclopramide*	*0.03–3*	*n/a*	*4.4–46*	*0.052*
8	Quetiapine	0.04–1	1.8–20	4–50	<0.002
9	Torasemide	1.1–18.5	n/a	n/a	<0.5
10	Escitalopram	0.01–1.7	0.48–5.9	3.2–49	0.047
11	Atorvastatin	0.007–0.25	n/a	n/a	0.084
*12*	*Diazepam*	*0.02–4.0*	*3–20*	*5–30*	*0.18*
*13*	*Oxazepam*	*0.1–1.4*	*>0.5*	*n/a*	*<0.002*
*14*	*Nordazepam*	*0.2–1.24*	*n/a*	*n/a*	*0.028*
*15*	*Temazepam*	*0.1–0.8*	*>1*	*n/a*	*0.006*
*16*	*Midazolam*	*0.02–0.5*	*0.2–2*	*2.4–62*	*0.006*
*17*	*Fentanyl*	*0.0003–0.01* (up to 0.04 **)	*0.003–0.02*	*0.003–0.21*	*0.003*
18	Tramadol	0.1–0.8	1–24	1.3–20	0.048
19	Zopiclone	0.02–1.3	0.25–1.6	0.4–4.1	0.012

* fatal intoxication with 4-MAA including the identified baclofen at the concentration of 106 µg/mL. ** tolerance may develop in chronic pain therapy (resulting higher blood levels).

**Table 4:** Reference values of pharmacologically active substances in urine compared to concentrations determined in patient’s urine collected postmortem (medical drugs with metabolites administered during hospitalization are marked in italics) [1,6,7].

No	Substance	Concentration in URINE, µg/mL			
		Therapeutical (Reference)	Toxic (Reference)	Lethal (Reference)	Patient (after Autopsy)
1	Pentoxifylline	n/a	n/a	0.8–7.9	22
2	Metformin	n/a	n/a	389	106
3	Acetaminophen (paracetamol)	n/a	n/a	180–1780	3.8
4	Metronidazole	n/a	n/a	n/a	0.92
*5*	*4-MAA (metamizole metabolite)*	*n/a*	*n/a*	*n/a*	*16*
6	Lidocaine	n/a	n/a	9–49	0.31
*7*	*Metoclopramide*	*n/a*	*n/a*	*n/a*	*0.54*
8	Quetiapine	n/a	n/a	3–151	<0.002
9	Torasemide	n/a	n/a	n/a	n/a
10	Escitalopram	n/a	n/a	0.5–276	0.19
11	Atorvastatin	n/a	n/a	n/a	n/a
*12*	*Diazepam*	*n/a*	*n/a*	*3*	*0.065*
*13*	*Oxazepam*	*n/a*	*n/a*	*1.3*	*0.004*
*14*	*Nordazepam*	*n/a*	*n/a*	*n/a*	*0.016*
*15*	*Temazepam*	*n/a*	*n/a*	*2.3*	*0.010*
*16*	*Midazolam*	*n/a*	*n/a*	*n/a*	*<0.002*
*17*	*Fentanyl*	*n/a*	*n/a*	*0.0002–0.8*	*<0.002*
18	Tramadol	n/a	n/a	n/a	0.27
19	Zopiclone	n/a	n/a	n/a	0.069

## Supplementary Materials

The following supporting information can be downloaded at: https://www.mdpi.com/article/10.3390/toxics10080447/s1.

## References

[1] Baselt R.C. (2014). Disposition of Toxic Drugs and Chemicals in Man.

[2] Suárez-Peñaranda J.M., Rico-Boquete R., López-Rivadulla M., Blanco-Pampín J., Concheiro-Carro L. (1998). A fatal case of suicidal pentoxifylline intoxication. Int. J. Legal Med..

[3] Sznajder I., Bentur Y., Taitelman U. (1984). First and second degree atrioventricular block in oxpentifylline overdose. Br. Med. J. (Clin. Res. Ed.).

[4] Eden G., Busch M., Kühn-Velten W.N., Schneider A., Kielstein J.T. (2011). Successful treatment of life-threatening pentoxifylline intoxication by high-flux hemodialysis. Clin. Nephrol..

[5] Bennis Y., Bodeau S., Batteux B., Gras-Champel V., Masmoudi K., Maizel J., De Broe M.E., Lalau J.D., Lemaire-Hurtel A.S. (2020). A Study of Associations Between Plasma Metformin Concentration, Lactic Acidosis, and Mortality in an Emergency Hospitalization Context. Crit. Care Med..

[6] Molina D.K., Hargrove V.M. (2019). Handbook of Forensic Toxicology for Medical Examiners.

[7] Moffat A.C., Osselton M.D., Widdop B. (2011). Clarke’s Analysis of Drugs and Poisons.

