# Supplementary material for: Fatal Suicidal Intoxication with Pentoxifylline Complicated by Cardiovascular Disorders
Source: Toxics. 2022 Aug 3;10(8):447. doi: 10.3390/toxics10080447 (PMC9414985; doi:10.3390/toxics10080447)
Supplement: Supplementary file 1 [file toxics-10-00447-s001.zip › toxics-1819168-supplementary.pdf]

Case Report

Electronic Supplementary Material - Fatal Suicidal Intoxication with Pentoxifylline Complicated by Cardiovascular Disorders

Jacek Sein Anand, Marek Wiergowski, Marek Roman Wiśniewski, Monika Kosmowska, Marzena Kata, Mateusz Kacper Woźniak

1. Materials

1.1. Chemicals and Reagents

Certified standards were purchased from the following manufacturers: LGC Standards (London, UK), Cerilliant (Round Rock, TX, USA), Chiron (Trondheim, Norway), and Cayman Chemical (Ann Arbor, MI, USA).

Pentoxifylline certified standard was produced by Dr. Ehrenstorfer GmbH and was obtained as a powder form in batch of 100 mg from LGC Standards (London, UK). Appropriate amount of the powder was dissolved in methanol (MeOH) to obtain a concentration of 1 mg/mL. Other standards were delivered as solutions in (MeOH) or in Acetonitrile (ACN) at concentrations of 0.1, 0.25, 1 or 2 mg/mL or in powder forms in batches of 1, 2, 5 or 10 mg. The powders were dissolved separately in MeOH to obtain a concentration of 1 mg/mL. These solutions were used as standard solutions for calibrations. Diazepam-D_5, prazepam-D_5, fentanyl-D_5, and quetiapine-D_8 were used as an internal standards (IS).

ACN, diethyl ether, ethyl acetate, MeOH, and formic acid (FA) were supplied by Sigma-Aldrich (Merck, Warszawa, Poland). All solvents used were of HPLC grade. Salts of analytical grade purity were purchased from POCH S. A. (Avantor Performance Materials Poland S.A., Gliwice, Poland). Phosphate buffer (pH 3.0) and carbonate buffer (pH 9.2 and pH 11) solutions were prepared by dissolving the appropriate salts in ultrapure water (resistivity of 18.2 MΩ*cm). Water was purified by a Millipore Synergy UV Elix 10 UV water system (Merck, Warszawa, Poland).

1.2. Biological Specimens

Biological materials used for the calibration consisted: drug-free (blank) blood samples (obtained from a regional blood donation bank (Gdańsk, Poland) collected from volunteers who were not consumers of any drug) and drug-free (blank) urine samples obtained from workers of our Department who were not consumers of any drug.

2. Instrumentations

2.1. GC-MS Analyses

Qualitative and quantitative analyses were performed using a 7890A GC System (gas chromatograph) equipped with a G4567A autosampler, a split/splitless injection port and a 5975C single quadrupole mass spectrometer (MS) with an electron ionization (EI) ion source (Agilent Technologies). The separation of analytes was carried out on a Phenomenex ZB-5 MS capillary column (30 m × 0.25 mm id, and 0.25 μm film thickness, Ship-pol, Izabelin, Poland) with helium at a purity of 99.999% as the carrier gas in a constant flow of 1 mL/min. The split injection mode (10:1) was used. Tthe injection volume was 2 μL. The oven temperature was programmed at 50°C for 1 min, then increased to 200°C at 20°C/min (held for 5 min), then ramped to 240°C at 10°C/min (held for 5 min) and finally ramped to 285°C at 10°C/min and held for 7 min. Post run conditioning was set at 285°C for 1 min. The temperatures of the injection port, MS transfer line, ion source and detector were set at 250, 285, 280 and 200°C, respectively. The MS was operated in positive mode (electron energy 70 eV). During qualitative analyses MS was operated in *SCAN* mode at *m*/*z* 35-380 while quantification was performed in *SIM* mode using ions listed in Table S2.

Data acquisition, result analysis and processing were performed using MSD ChemStation E.02.02.1431 software by Agilent Technologies, Inc.

2.2. HPLC-UV-VIS/DAD Analyses

Qualitative analyses were performed using the high-performance liquid chromatography (HPLC) LC-10ADvp system (Shimadzu, Japan) comprising a DGU-14ALvp degasser, a SLC-10Avp controller, a FCV-10ALvp binary pump, and an SIL-10AF autosampler. Separation was achieved on a monolithic Chromolith® Performance RP-18e HPLC column (100 × 4.6 mm) equipped with a Chromolith® RP-18e guard cartridge (10 × 4.6 mm) (Merck KGaA, Warszawa, Poland). The column was not thermostatted (it was maintained at room temperature), the flow rate was kept at 1mL/min, and the injection volume was 20 μL. The mobile phase used for the separation consisted of water with 0.01% phosphoric acid (*v/v*; component A) and ACN (*v/v*; component B). Chromatographic separation was performed in gradient elution from 0% B to 100% B at 20 min. Then, the initial column conditions were restored over 6 min.

The detection system consisted of a SPD-10AVvp UV-VIS detector and a SPD-M10Avp dioda-array detector (DAD) by Shimadzu, Japan. DAD was operated in the range from 190 nm to 370 nm. Data acquisition, result analysis and processing were performed using LabSolutions software by Shimadzu Corporation (version 5.93).

2.3. LC-MS/MS Analysis

Analyses were performed on an Agilent Technologies 1260 Infinity liquid chromatograph including an online degasser, a binary pump, and an autosampler coupled to a 6420 Triple Quad mass spectrometer (Santa Clara, CA, USA). Chromatographic separation of the analytes was achieved on an Agilent Technologies InfinityLab Poroshell 120 EC-C18 column (3.0 mm × 100 mm × 2.7 μm) equipped with an InfinityLab Poroshell 120 EC-C18 Fast Guard (3.0 mm × 5 mm × 2.7 μm) as a pre-column. Mobile phase consists of 0.05% FA in water (*v/v*, component A) and 0.05% FA in ACN (*v/v*, component B). The flow rate of the mobile phase was set to 0.5 mL/min. Data acquisition, result analysis and processing were performed using MassHunter software by Agilent Technologies (version B.08.00).

ESI–MS/MS-detection (electrospray ionization) was performed in multiple reaction monitoring (MRM) mode using positive ionization (ESI+). The two most abundant MRM transitions were chosen for each compound: the first for quantification and the second for qualification, Optimization of the MRM transitions and corresponding collision energies (CEs) and fragmentor voltages was performed using the MassHunter Optimizer by Agilent Technologies. Source parameters were optimized by the use of Source Optimizer software (Agilent Technologies).

In method 1 (Table S1) chromatographic separation was performed in gradient elution mode as follows: 0 min (10% B), 1 min (10% B), 6 min (70% B), and 7 min (95% B) kept for 3.5 min. Then, the initial column conditions were restored over 4 min. The column was thermostatted at 30°C. Injection volume was 5 μL. Source parameters were set as follows: gas flow (nitrogen, 5.0 purity): 10 L/min, gas temperature: 350°C, nebulizer pressure: 50 psi, and capillary voltage: 3500 V.

In method 2 (Table S1) chromatographic separation was performed in gradient elution mode as follows: 0 min (10% B), 0.5 min (10% B), and 10 min (95% B) kept for 3 min. Then, the initial column conditions were restored over 4 min. The column was thermostatted at 35°C. Injection volume was 10 μL. Source parameters were set as follows: gas flow (nitrogen, 5.0 purity): 10 L/min, gas temperature: 350°C, nebulizer pressure: 50 psi, and capillary voltage: 4000 V.

3. Details of Methods Used for Quantification

Analytes isolation methods used for qualification consisted:

1.extraction in acidic condition – LLE, 500 μl of sample, pH 3, 1 mL of diethyl ether, 15 min mixing,

2.extraction in basic conditions – LLE, 500 µl of sample, pH 11, 1 mL of ethyl acetate, 15 min mixing.

Quantification was performed by methods described in Table S1 (presented below) using parameters presented in section 2.1 and 2.3. It should be pointed out, that method 1 and 2

Table S1. Description of methods used for quantification.

No. of method	Compound	Extraction	Internal standard (IS)	Analytical methods
1	Pentoxifylline, Metformin, Acetaminophen (paracetamol), Metronidazol, Lidocaine, Metoclopramide, Quetiapine, Torasemide, Escitalopram, Atorvastatin	Protein precipitation (as described in section 5.1).	Prazepam-D_5, Quetiapine-D_8 (for quetiapine qiantifiction)	LC-MS/MS
2	Diazepam, Oxazepam, Nordazepam, Temazepam, Midazolam, Fentanyl, Tramadol, Zopiclone	LLE: 500 µl of sample, pH 9.2, double extraction with1 mL of ethyl acetate (mixing 1 min after each portion of solvent).	Diazepam-D_5 and and Fentanyl-D_5 (for fentanyl quantification).	LC-MS/MS
3	4-MAA	LLE: 500 µl of sample, pH 11, 1 mL of ethyl acetate, 15 min mixing.	Prazepam-D_5	GC-MS

4. Analytical parameters

All analytical/chromatographic parameters were carefully optimised. Selected parameters used for quantification were presented in Table S2.

Table S2. Retention times and parameters for MRM/SIM mode for the studied compounds and the IS. Bolded MRM transitions and ions were used for quantification.

Compound	MRM/SIM	CE [V]	CAV [V]	Frag [V]	Rt [min]
Pentoxifylline	**279.1→ 181.1**<br>279.1→ 38.1	20<br>20	3	150	5.72
Metformin	**130.1→ 60.1**<br>130.1→ 71.1	10<br>20	3	150	0.99
Acetaminophen (paracetamol)	**152.1→ 110.1**<br>152.1→ 65.0	10<br>40	3	150	2.09
Metronidazole	**172.1→ 128.0**<br>172.1→ 82.1	10<br>20	3	120	2.29
Lidocaine	**235.2→ 86.1**<br>235.2→ 58.2	16<br>36	4	118	5.12
Metoclopramide	**300.1→ 227.1**<br>300.1→ 184.0	20<br>40	3	150	5.34
Quetiapine	**384.2→ 253.2**<br>384.2→ 221.1	20<br>44	4	161	6.35
Torasemide	**349.1→ 264.1**<br>349.1→ 183.1	20<br>40	3	150	6.39
Escitalopram	**325.2→ 109.1**<br>325.2→ 262.2	17<br>33	4	130	6.59
Atorvastatin	**559.3→ 440.2**<br>559.2→ 250.1	20<br>40	3	150	8.48

Diazepam	**285.1→ 193.1**<br>285.1→ 154.0	37<br>25	5	140	8.7
Oxazepam	**287.1→ 241.1**<br>287.1→ 269.1	9<br>21	5	140	7.5
Nordazepam	**271.1→ 140.0**<br>271.1→ 165.0	29<br>29	5	50	7.9
Temazepam	**301.1→ 255.1**<br>301.1→ 283.1	21<br>9	5	50	8.2
Midazolam	**326.1→ 291.1**<br>326.1→ 209.1	29<br>37	5	50	6.5
Fentanyl	**337.2→ 105.0**<br>337.2→ 188.0	40<br>24	3	146	6.5
Tramadol	**264.2→ 58.2**<br>264.2→ 42.2	16<br>60	4	118	5.2
Zopiclone	**389.1→ 245.0**<br>389.1→ 217.0	12<br>36	4	93	5.4
4-MAA	**56**, 83, 217	-	-	-	14.80
Prazepam-D_5 (method 1)	**330.1→ 276.1**<br>330.1→ 140.0	20<br>40	3	150	8.96
Quetiapine-D_8	**392.2→ 258.1**<br>392.2→ 257.1	25<br>21	5	170	6.31
Diazepam-D_5	**389.1→ 198.1**<br>389.1→ 154.0	40<br>40	3	150	8.6
Fentanyl-D_5	**342.3→ 105.1**<br>342.3→ 188.1	40<br>20	3	150	6.4
Prazepam-D_5 (method 3)	**269**, 296, 91	-	-	-	25.66

CE [V] – collision energy, CAV [V] – cell accelerated voltage, Frag [V] – fragmentor voltage, Rt [min] – retention time.

5. Quantification and Calibration Data

Quantification and calibration were performed for blood and urine. It is important that all presented data are parts of our in-house developed methods used in routine toxicological analyses which contains more compounds than listed in this paper, e.g. method 2 allows for quantification of total 54 benzodiazepines and opioids. However, for the purpose of this paper we focused on data for pentoxifylline.

5.1. Sample Preparation

To the blood sample (0.2 mL) placed in 1.5 mL Eppendorf vials, 10 µl of a mixture of IS at a concentration of 50 µg/mL (also containing praepam-D_5) was added. The sample was precipitated with ACN. Six hundred microlitres of acetonitrile were added in 100 uL portions, and after each addition, the sample was vortex mixed for 10 s. The sample was centrifuged at 14500 rpm for 2 min. The upper layer was than transferred to a 5 mL glass vial and acetonitrile was evaporated to dryness under nitrogen at 40°C. The dry residue was dissolved in 100 µL of mobile phase (water:ACN, 1:9, *v*/*v*) and transferred to inserts for autosampler vials.

5.2. Calibration

Matrix-matched calibration of the LC-MS/MS was performed using the internal standard method. Calibration standards (number of replicates n = 3) were prepared by spiking of 0.2 mL of blank blood and urine samples with adequate amounts of pentoxifyilline standard solution in a range of 0.5 – 10 µg/mL. Then, whole extraction procedure

was performed. A five-point calibration curves (0.5, 1.25, 2.5, 5 and 10 μg/mL) based on the peak area ratios of the analytes to the IS against nominal analyte concentration were built. Weighted least squares regression (1/*x*) was used to improve accuracy, especially in low calibration levels. Due to use wide calibration range, quadratic type of calibration curve was used for blood. Such approach allowed to obtain good accuracy. Linearity was expressed as the correlation coefficient (*r*) of the constructed calibration curves. Limit of quantification (LOQ) generally corresponds to the lowest point on the calibration curve that is linear, but verification of a *S*/*N* ratio equal to minimum 10 with imprecision within 20% was also performed.

Calibration curves were linear in studied concentration ranges with *r* over 0.99. Accuracy was in the range of 85-115% what is appropriate for bioanalytical methods. Recovery of extraction was not investigated because matrix-matched calibration was selected in the study what minimize matrix effects. Protein precipitation as a sample preparation method was found to be appropriate for quantification of pentoxifylline in blood and urine samples with high sensitivity. LOQ could be even lower and calibration range wider, however we selected such calibration range to met concentration level of pentoxifylline in real samples and/or in therapeutic cases. Proper linearity was also taken into account in chosen of a calibration range. In case of urine samples, the concentration of pentoxyfilline was above the range of the calibration curve. Therefore, the samples were diluted with drug-free urine (the same urine that was used for the calibration) to perform the quantification within the ranges of the calibration curves.

Summary of quantification and calibration data for pentoxifyilline are presented in Table S3.

Table S3. Quantification and calibration data for pentoxifyilline.

Samples	Calibration range [μg/mL]	Numbers of calibration points	Linearity type	Calibration curve				Weighting factor	Accuracy [%]	LOQ [μg/mL]
				a	*b*	*c*	*r*			
**Blood**	**0.5 - 10**	**5**	**quadratic**	**-3.0e-8**	**0.0013**	0.1868	0.9936	1/*x*	85.5-113.5	0.5
Urine	0.5 - 10	5	Linear	0.00118	-0.0667	-	0.9972	1/*x*	85.6-108.8	0.5
